# Epidemiology of invasive meningococcal disease in Cyprus 2004 to 2018

**DOI:** 10.2807/1560-7917.ES.2020.25.30.1900534

**Published:** 2020-07-30

**Authors:** Maria Koliou, Diamanto Kasapi, Stella Mazeri, Panagiota Maikanti, Anna Demetriou, Chrystalla Skordi, Maria Agathocleous, Georgina Tzanakaki, Elisavet Constantinou

**Affiliations:** 1 Medical School, University of Cyprus, Nicosia, Cyprus; 2 Unit for Surveillance and control of Communicable diseases, Medical and Public Health Services, Ministry of Health, Nicosia, Cyprus; 3 The Royal (Dick) School of Veterinary Studies, The University of Edinburgh, Edinburgh, United Kingdom; 4 The Roslin Institute, Division of Genetics and Genomics, Easter Bush Veterinary Centre, Roslin, United Kingdom; 5 Department of Microbiology, Nicosia General Hospital, Nicosia, Cyprus; 6 Health Monitoring Unit, Ministry of Health, Nicosia, Cyprus; 7 Larnaca General Hospital, Larnaca, Cyprus; 8 Paediatric Department, Limassol General Hospital, Limassol, Cyprus; 9 National Meningitis Reference Laboratory, Department of Public Health Policy, School of Public Health, University of West Attica, Athens, Greece; 10 Medical and Public Health Services, Ministry of Health, Nicosia, Cyprus

**Keywords:** Invasive meningococcal disease (IMD), Neisseria meningitidis, Epidemiology, risk factors, immunisation programmes, Cyprus

## Abstract

**Background:**

Despite progress in the management of invasive meningococcal disease (IMD) it causes significant mortality and sequelae.

**Aim:**

This study aims to describe the epidemiology and clinical characteristics of IMD in Cyprus and discuss the current immunisation programmes.

**Methods:**

This is a retrospective study of all cases of IMD notified to the Ministry of Health between 2004 and 2018. Demographic, epidemiological, clinical and microbiological data were collected when a new case was notified. Risk factors associated with mortality were investigated using univariable logistic regression.

**Results:**

54 cases of IMD were recorded, an overall incidence of 0.4 cases per 100,000 population. The incidence rate was highest among infants (7.2/100,000) and adolescents (1.4/100,000). Case fatality rate was 10.4%. Serogroup B accounted for 24 of 40 cases caused by known serogroup. Serogroups W and Y comprised nine cases and were responsible for most fatal cases. Serogroup C was the cause in only four cases. There was an increase in the odds of death with increasing age, while the presence of meningitis in the clinical picture was found to be associated with lower odds of death.

**Conclusion:**

Despite the low incidence of IMD in Cyprus, it remains an important cause of morbidity and mortality. Serogroup B is the most frequent serogroup, while incidence of serogroups W and Y is rising. Monitoring new cases and yearly evaluation of the immunisation programmes by the National Immunization Technical Advisory Group (NITAG) is essential for successful control of the disease.

## Introduction

Meningococcal disease is a potentially severe bacterial infection caused by *Neisseria meningitidis,* often referred to as meningococcus. *Neisseria meningitidis* colonises the nasopharynx of healthy individuals. The frequency of colonisation is ca 5–10% in healthy adults and up to 25% in adolescents [1]. In addition to colonisation, *N. meningitidis* can cause invasive disease, the most frequent forms of which are meningitis or bacteraemia/septicaemia or both [2,3]. Depending on the antigen of the polysaccharide capsule, meningococci are classified in 12 different serogroups. The most common serogroups are A, B, C, W, X and Y [3]. In Europe and the Americas, the most frequent serogroups causing disease are B and, less frequently, C, W and Y, whereas in Africa, A and X are most common [4,5].

Between 2004 and 2014, 49,269 meningococcal disease cases were recorded in European countries [5]. The mean annual incidence across Europe was 0.9 cases per 100,000 population. The highest annual incidence, 16 of 100,000, was found in infants younger than 1 year. Increased numbers of cases were also found in the age groups 1–4 years (4.9/100,000) and 15–24 years (1.4/100,000). The most frequent serogroups were B and C, which represented 74%, and 16% of all cases, respectively. Nevertheless, in recent years, B and C serogroups have followed a downward trend in many countries. In the European Centre for Disease Prevention and Control (ECDC) annual epidemiological report for 2017, serogroup B caused 51% of cases. An threefold increase was noted in the incidence of serogroup W invasive meningococcal disease (IMD) between 2013 to 2017. Serogroup Y represented 12% of the cases in 2017 [6].

The severity of meningococcal disease lies in the high mortality rates, which range between 5% and 15%, and the high risk of complications in survivors [7,8].

Different studies have reported risk factors associated with fatal outcome. Characteristically, high bacterial load is related to high mortality rate and high complication rates in the survivors [9]. Some other studies have further associated mortality by IMD with increasing age, the presence of septicaemia without meningitis and a period of less than 24 h between onset of symptoms and admission to hospital [10,11].

Introduction of vaccination against the different serogroups has significantly decreased the incidence of the disease. The first country to introduce the vaccine against serogroup C (MenC conjugate vaccine) was the United Kingdom (UK) in 1999. This intervention led to a decrease of more than 90% in laboratory-confirmed cases caused by serogroup C in immunised age groups [12,13]. Cases also decreased in other age groups by around two thirds as a result of reduction in carriage [14]. Similar decreases in the frequency of serogroup C disease were detected in a number of European countries who had introduced MenC vaccination in their national immunisation programmes [5]. Since 2009, there has been an increase in disease caused by serogroups W and Y in a number of European countries and the United States (US) [15,16]. Therefore, the quadrivalent conjugate vaccine against serogroups A, C, W and Y (MenACYW) replaced the MenC vaccine and has been administered mainly in adolescence in some countries [2,17].

Following the dramatic decrease in cases caused by serogroup C, serogroup B has become the most frequent cause of meningococcal disease in most European countries. In European countries, serogroup B represented 67.8% of laboratory-confirmed cases in 2012 and 51% in 2017 [6,18]. In the UK between 2006/07 and 2010/11, it accounted for 87% of meningococcal disease cases, with the highest incidence among infants [19]. In September 2015, the UK was the first country to introduce a universal infant immunisation programme against serogroup B (4CMenB protein vaccine). This programme has led to a significant decrease in IMD attributable to serogroup B, with an effectiveness of 82.3% in infants younger than 12 months [20].

During our study period, two different sectors of healthcare providers were operating in Cyprus, the public sector and the private sector, each with its own immunisation regimen, the public sector serving around 60% of the toddler population [21]. As a result, the MenC conjugate vaccine was introduced in two phases. In the private sector, it was introduced in early 2001, while in the programme of the public sector, free of charge to the whole population, it was introduced much later, at the end of 2006. According to the 3-yearly national vaccination coverage survey in toddlers, vaccination coverage of 17–24 months-old toddlers increased to high levels only after 2006 [22]. More precisely, the vaccination coverage for MenC vaccine increased from 49.2% in 2006 to 84.8% in 2012 and since then, coverage rate in toddlers has remained high [21]. In 2014, the private sector replaced the MenC vaccine given in infancy with MenACYW, given in one dose at the age of 12 months. More recently, in January 2017, the new 4CMenB vaccine was also introduced but only in the private sector. Since 2019, both immunisation schemes have been integrated and MenACWY is given to all population at the age of 12 months.

In Cyprus, there are no published epidemiological data on the incidence and mortality of meningococcal disease in the population except as part of the annual reports by the ECDC [23]. This study aimed to describe the epidemiology and the characteristics of IMD cases in Cyprus, including the most prevalent serogroups causing disease, and to study the association of several risk factors with a fatal outcome. It also discusses and evaluates the current immunisation programmes operating in Cyprus.

## Methods

### Study area

This is a retrospective study of all cases of IMD diagnosed in Cyprus (the Government-controlled area), between 1 January 2004 and 31 December 2018.

The population of Cyprus between 2004 and 2018 ranged from 733,067 in 2004 to 854,802 at end of 2016 [24]. 

### Definitions

The EU case definition defines a confirmed case of IMD as any case that fulfils the laboratory criteria for diagnosis of IMD. These are any of the following: isolation of *N. meningitidis* from a normally sterile site or from purpuric skin lesions or detection of nucleic acid of *N. meningitidis* from a normally sterile site or from purpuric skin lesions or detection of *N. meningitidis* antigen or Gram-negative diplococci in cerebrospinal fluid (CSF) [25]. Because the *N. meningitidis* antigen test does not discriminate between serogroup W and Y, cases of W and Y are sometimes processed together as W/Y in this manuscript.

Clinically compatible cases refer to cases with typical clinical features strongly suggestive of meningococcal disease such as fever, stiff neck, petechial or purpuric rash, septic shock and/or CSF findings suggestive of meningitis, but without bacteriological confirmation [26].

For further analysis of the cases, the presence of bloodstream infection or meningococcaemia/septicaemia was defined as a positive blood culture and/or positive blood PCR test [27].

Presence of meningitis only was defined as the clinical picture of meningitis without the typical rash but with positive cultures/PCR, Gram stain or rapid antigen test for *N. meningitidis* from CSF [26].

### Surveillance of invasive meningococcal disease

In Cyprus, surveillance of IMD is based on the mandatory notification system as every practicing physician who diagnoses a new case of IMD must notify the public health authorities by completing a notification form. The operational body responsible for processing all information on communicable diseases is the Unit for Surveillance and Control of Communicable Diseases (USCCD). This Unit administratively belongs to the Directorate of the Medical and Public Health Services of the Ministry of Health. In addition to confirmed cases, each physician has to notify each suspect case by telephone directly to the staff of the Unit within 24 h of examining the patient, so that appropriate public health measures are promptly taken. The notification form collects demographic data on the case such as age, sex, residence, any epidemiologic link to other cases and whether the case was imported.

All invasive strains isolated in regional laboratories were referred to the Microbiology Department of Nicosia General Hospital where serogrouping of each isolate is performed. Meningococcal isolates were cultured on Chocolate Columbia Agar (OXOID Ltd, Basingstoke, UK) and incubated at 37 °C and 5–10% CO_2_ for 24 h. Serogroups were identified by a slide agglutination test (Remel Europe Ltd., Dartfort, UK) [28]. The laboratory of the Nicosia General Hospital notified the serogroup results both to the USCCD and to the clinicians who manage the IMD case. Isolates were then sent to the National Meningitis Reference Laboratory in Athens, Greece for further molecular typing. Furthermore, for culture-negative suspected biological samples, a multiplex PCR assay was carried out for identification and typing of *N. meningitidis* [29]. Genogroup was determined by multiplex PCR targeting specific capsule group genes, as described previously [30].

All meningococcal isolates belonging to a sero-/genogroup were characterised by finetyping (multilocus sequence typing and PorA and FetA typing), as described previously [31], using the PubMLST.org/neisseria database (http://pubmlst.org/ neisseria/). Sequence types (ST) were defined and grouped into clonal complexes (ccs). PorA genotyping for variable regions 1 and 2 (VR1 and VR2) was performed as described previously [32] and compared with the variable sequences in the *Neisseria* PorA database (http://pubmlst.org/neisseria/PorA/). Similarly, the FetA variable region was also obtained for all typeable isolates, as previously described [33] and compared with variable sequences in the *Neisseria* FetA database (http://pubmlst.org/neisseria/FetA/). All results are submitted to The European Surveillance System (TESSy) on an annual basis.

In 2017, a molecular method was also introduced at the microbiology department of Nicosia General Hospital, for the detection of *N. meningitidis* and other pathogens in CSF specimens. The method is an automated multiplex RT-PCR, using the Film Array Meningitis/Encephalitis (ME) Panel Analyzer (BIOFIRE diagnostics, LLC, Salt Lake City, US) applied for detecting pathogens directly from CSF specimens. The method only detects species and not serogroups or other characteristics [34].

The CSF specimens from cases from all districts in the Government-controlled area of Cyprus which are clinically compatible with bacterial meningitis are sent to this laboratory in order to be processed by this technique. Since the introduction of this new methodology, the yield of detection of meningococcal disease has further increased, particularly in culture-negative cases. The CSF specimens are referred for molecular diagnostics before the result of traditional culture is known. Therefore, in some cases there may be positive molecular as well as traditional culture results for a CSF specimen.

When an IMD case is laboratory-confirmed, a second more extensive questionnaire on clinical and laboratory findings of the case is completed and sent to the USCCD by the diagnosing physician. This questionnaire includes details on the clinical picture on admission as well as the laboratory findings in blood and CSF and information on the outcome of the case.

### Data collection

For the purposes of this study, data were collected from (i) the notification forms and the extensive questionnaires on confirmed and clinically compatible cases of IMD kept at the USCCD and (ii) additional data were retrieved from patients’ records in the public hospitals in cases of existing gaps in clinical information. Data between 1998 and 2003 on numbers of cases each year were also retrieved from notification forms kept at the USCCD.

### Study subjects

Patients hospitalised between 1 January 2004 and 31 December 2018 and diagnosed as confirmed or clinically compatible cases of IMD were included in this study. These patients were hospitalised in all five public and the two largest private hospitals in Cyprus. The catchment area of these hospitals is the whole population residing in the Government-controlled area of Cyprus.

### Statistical analysis

Data were analysed using the R statistical software version 3.5.1 [35]. Package ggplot2 was used for all plots [36]. Univariable logistic regression was used to investigate the association between patient-related risk factors and death. Models were fit using Firth's bias reduction method in package logistf to address issues of separation [37,38].

### Ethical statement

The National Bioethics committee granted exemption to this study as this was a retrospective study where all data were used anonymously and special care was taken to preserve the protection of personal data.

## Results

**Figure 1:**
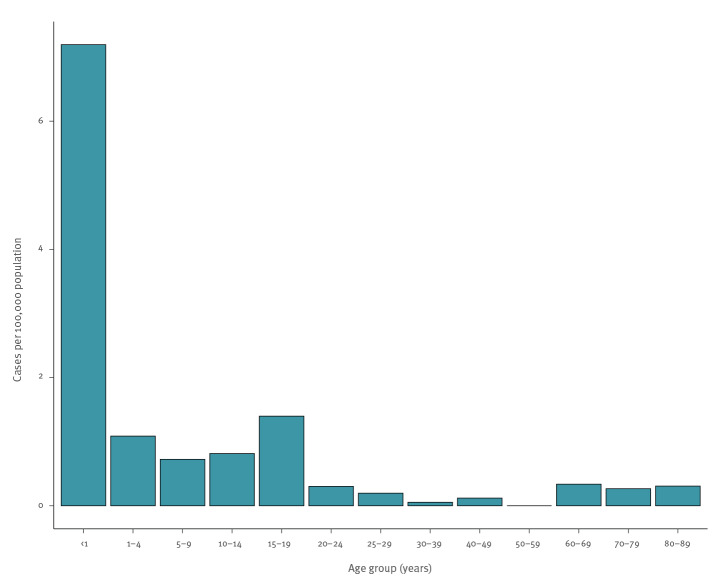
Incidence rate of meningococcal disease by age group, Cyprus, 2004–2018 (n = 54)

Between 1 January 2004 and 31 December 2018, 54 cases of IMD were reported to the health authorities of Cyprus. A total of 25 cases were male and 29 cases were female, with an age range from 19 days to 89 years. The mean age of patients was 19.6 years and the median age was 14.5 years. Four of these cases were compatible with a strong clinical suspicion for IMD, but without laboratory confirmation. In two of the notified cases, there was no information on the method of diagnosis. A total of 41 of the 54 cases occurred in Cypriot citizens, while the remainder were British citizens (n = 7), Eastern European nationals (n = 4), Greek (n = 1) and Swedish (n = 1). Seven cases were imported. No secondary cases were reported. The overall mean annual incidence rate was 0.4 cases per 100.000 population, ranging from 0.1 in 2011 to 0.8 in 2005 and 2012. The incidence rate was highest among children younger than 1 year, reaching 7.2 cases per 100,000 population in this age group (10 cases). A second peak was noted in adolescents aged 15–19 years with 1.4 cases per 100,000 population (12 cases). The lowest incidence was observed in 30–59 year-olds (0.06 cases per 100,000 population; three cases). Figure 1 shows the distribution of IMD by age group.

**Figure 2:**
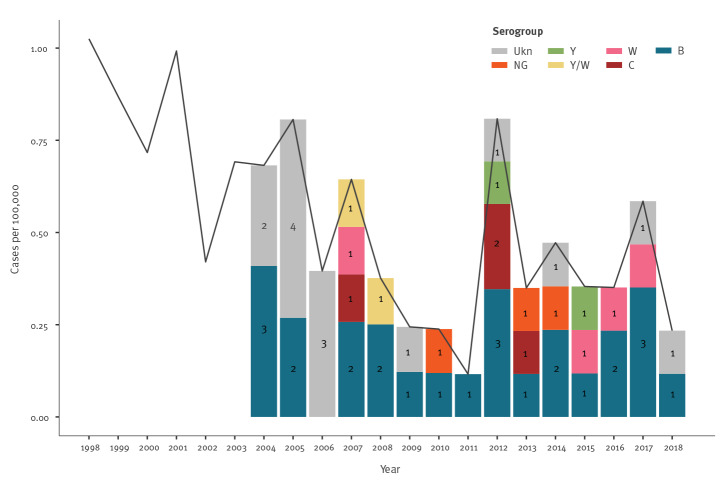
Incidence of meningococcal disease, Cyprus, 1998–2018 (n = 87)

NG: non-groupable; Unk: unknown (samples for which the isolate was not sent to the reference laboratory for serogrouping.

As shown in Figure 2, the incidence of meningococcal disease in Cyprus decreased between 1998 and 2011. However, after a peak in 2012, the mean incidence appeared to have reached a higher plateau, fluctuating between 0.2 and 0.6 cases per 100,000.

Bars: distribution of serogroups among cases for each year; black line: total number of cases for each year. The individual number of cases per serogroup is indicated on the bars.

Serogroup B was the most frequent serogroup causing disease, accounting for 24 of 40 cases with known serogroup (Figure 2). Moreover, serogroup B represented an even higher proportion of cases in age groups with a higher incidence of meningococcal disease: five of seven cases in infants, eight of 11 cases in children younger than 5 years and seven of 10 cases in 15–19 years-olds. Serogroup C accounted for only four of 40 cases overall, similar to serogroup W. Overall, serogroups W and Y together accounted for a total of eight of the 40 cases and for three of the four fatalities that were caused by strains with known serogroup.

Serogrouping was not performed before 2004. Even between 2004 and 2011, unknown serogroups were recorded in 10 of 27 cases. In these cases, the isolates were not sent to the reference laboratory for serogrouping (Figure 2). However, between 2012 and 2018, unknown serogroups were reported in only four of 27 cases of IMD.

### Seasonal distribution

**Figure 3:**
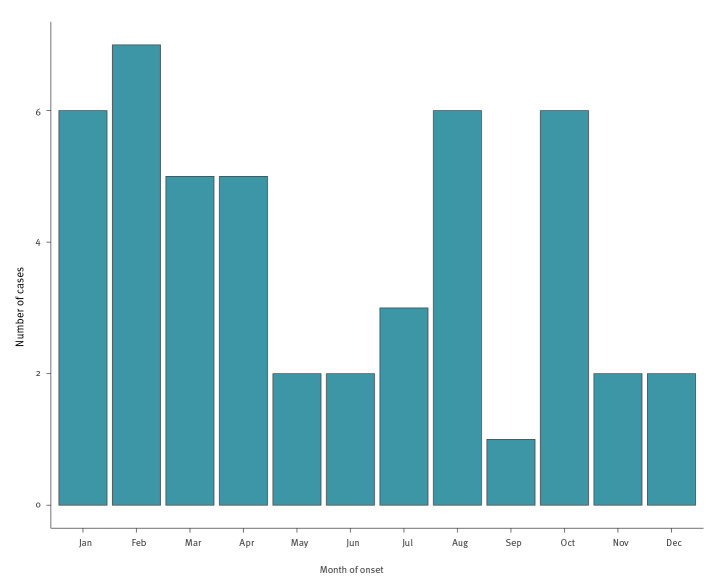
Month of onset of local cases, Cyprus, 2004–2018 (n = 47)

Figure 3 shows the distribution of all cases per month after exclusion of the imported cases. While cases were spread throughout the year, a large proportion of them (23/47) occurred between January and April.

Imported cases (n = 7) are not included in the figure.

### Clinical characteristics

Table 1 shows the epidemiological as well as clinical characteristics of cases. The ratio of male to female cases was 1:1.2. Nearly half of the cases for whom this information was available were classified as both meningitis and septicaemia (25/53), while 16 cases were diagnosed as only meningitis and 12 cases as only septicaemia. Culture of sterile body fluids such as blood or CSF confirmed the diagnosis in 31 of 52 cases (Table 1) and molecular methods (PCR) in nine cases. One case was diagnosed by both culture and PCR. No cases were diagnosed by culturing the skin lesions. Five cases were diagnosed by CSF latex agglutination and two cases by CSF Gram stain. Four cases were only diagnosed based on the clinical picture as compatible with IMD.

**Table 1:** Characteristics of cases of invasive meningococcal disease, Cyprus, 2004–2018 (n = 54)

Characteristics	Category	Number	%
Sex	Male	25/54	46.3
	Female	29/54	53.7
Clinical picture^a^	Meningitis	16/53	30.2
	Septicaemia	12/53	22.6
	Meningitis and septicaemia	25/53	47.2
Outcome^b^	Death	5/48	10.4
	Recovered	43/48	89.6
Method of diagnosis^c^	Culture	31/52	59.6
	PCR	9/52	17.3
	Culture and PCR	1/52	1.9
	Latex agglutination^d^	5/52	9.6
	CSF Gram stain	2 /52	3.8
	Clinical diagnosis	4/52	7.7

CSF: cerebrospinal fluid.

^a^ In one case, the clinical picture (i.e. meningitis or septicaemia) was unknown.

^b^ Outcome was unknown for six cases.

^c^ In one case, both culture and PCR were positive. In two cases, no information was given about the method of diagnosis.

^d^ In cases of positive latex agglutination in CSF, serogroup Y cannot be discriminated from serogroup W. Therefore, the two serogroups were counted together.

### Risk factors for death

Among 48 cases with known outcome, five patients died (10.4%). Results of the univariable logistic regression investigating factors associated with death are shown in Table 2. This analysis found no statistically significant association between sex and death (p = 0.219). On the other hand, when age was used as a numerical dependent variable, there was an increase in the odds of death with increasing age, which was statistically significant (p = 0.050). The presence of meningitis vs meningococcaemia decreased the odds of death 17 times (p = 0.007). In terms of factors relevant to the clinical presentation of the patients, time from onset of symptoms to admission, white blood cell count on admission and lethargy or confusion on admission were examined, but no statistically significant associations were found. Y or W serogroup were the cause of the disease in three of four fatal cases where the serogroup was determined and the association between these serogroups and death was statistically significant (p = 0.032).

**Table 2:** Univariable logistic regression analysis of possible risk factors for death in invasive meningococcal disease cases, Cyprus, 2004–2018 (n = 54)

Risk factor	Odds ratio	95% confidence interval	p value
Presence of meningitis			
No	Ref		
Yes	0.08	0.01–0.61	0.007
Age (years)	1.03	1.00–1.07	0.050
Sex			
Female	Ref		
Male	0.32	0.04–2.31	0.219
White blood cell count on admission			
High	Ref		
Normal/low	3.54	0.45–27.9	0.198
Unknown	4.27	0.32–56.21	0.256
Confusion or lethargy on admission			
No	Ref		
Yes	7.34	0.33–162.09	0.116
Unknown	43.00	1.33–1389.16	0.008
Onset of symptoms to admission < 24 h			
No	Ref		
Unknown	17.00	0.67–434.16	0.053
Yes	3.60	0.46–28.11	0.190
Serogroup			
B	Ref		
C	1.59	0.01–35.55	0.793
W or Y	9.12	1.21–110.02	0.032
Non-groupable	2.87	0.02–73.39	0.578
Unknown	1.87	0.14–25.09	0.608

Ref: reference category.

## Discussion

In this study we describe and analyse the epidemiology and clinical characteristics of IMD in Cyprus. The mean incidence of IMD in Cyprus was estimated at 0.4 cases per 100,000 population over a period of 15 years. This estimate is lower than for some other Mediterranean countries such as Malta or France and similar to the incidence in Greece and Spain [39]. However, the incidence in infants was much higher and was the highest of all age groups. This is the case in many countries and is believed to be the result of their insufficiently developed immune system and the gradual loss of the antibodies acquired via the placenta [40]. A second high peak in incidence was noted in adolescents, which is in agreement with the epidemiology of IMD in most other countries [5].

The incidence of meningococcal disease in Cyprus decreased between 1998 and 2011. However, after a peak in 2012, the mean incidence appeared to have reached a higher plateau. This small upward trend in the incidence of cases after 2012 may have partly been due to increased awareness among physicians of their responsibility to notify cases. During that year, the USCCD ran an awareness campaign directed at physicians, which included talks at several medical conferences. At the same time, national guidelines were issued on the notification and laboratory investigation of cases. Further to this, the Reference Laboratory at the Nicosia General Hospital employed more sensitive molecular techniques for the detection of pathogens in new cases after 2011. Before 2017, specimens were sent to the Meningococcal Reference Laboratory in Athens, Greece for molecular diagnosis of suspect IMD disease. In 2017, the RT-PCR technique was applied at the Microbiology Reference Laboratory in the Nicosia General Hospital. It has been well recognised in several studies that the use of PCR increases diagnostic sensitivity when compared with traditional culture of blood or CSF alone. In a study from Ireland, blood cultures were positive in only eight of 39 PCR-confirmed cases, partly because of the administration of antibiotic treatment before cultures were taken [41]. In the UK, 58% of cases were confirmed by PCR only [42].

During the study period in Cyprus serogroup C was a rare cause of meningococcal disease restricted in single cases and no more cases were detected after 2013. A decrease in serogroup C disease was also noted in other European countries as a result of the high MenC vaccine coverage in children [5,12].

Serogroup B appeared to be the most frequent serogroup causing disease in the population of Cyprus especially in infants and adolescents. This is the case as in many other European countries [5]. The National Immunization Technical Advisory Group (NITAG) assessed the potential introduction of meningococcal B vaccine into the national immunisation programme at the end of 2018. Based on various factors such as the low incidence of meningococcal disease in Cyprus so far, it was decided to offer immunisation with MenB at this stage only to groups at high risk for severe meningococcal disease, such as people with asplenia and other immunocompromising conditions. This decision is compatible with the one taken by NITAGs in many other European countries thus far [43]. The possible introduction of the vaccine will be reevaluated in 2020, as new scientific data come to light.

Serogroups W and Y appeared to cause a considerable proportion of cases of meningococcal disease. The recent increase in these two serogroups was also noted in other European countries [15,16]. In addition, these two serogroups were associated with a higher risk of death and accounted for three of the four fatal cases caused by a known serogroup. Therefore, the NITAG decided in 2019 that the MenACWY vaccine will be given in the national immunisation schedule at the age of 12 months instead of the monovalent MenC vaccine, and also to male adolescents at the age of 18 years before joining the armed forces. MenACYW is also given to prospective students at colleges or universities in Cyprus, in other European countries and in the US [44,45].

A substantial proportion of cases occurred in winter and the early spring months, especially between January and April. The increased seasonal incidence in winter months was in agreement with the observed epidemic waves in the past i.e. between 1936 and 1937. At that time, large epidemic waves of IMD in Cyprus were associated with cold wet weather, which was considered to result in overcrowding indoors. Epidemics of seasonal influenza were also found to be associated with the peak of the epidemic waves of IMD [46]. Respiratory viruses in general and influenza viruses specifically have been associated with increased numbers of IMD cases [47]. It has been reported that influenza A virus neuraminidase may have a potential role in facilitating meningococcal adhesion to influenza virus-infected epithelial cells through interaction with sialic acid-containing meningococcal capsules [48].

The case fatality ratio for IMD in Cyprus remains substantial as is the case in most other high-income countries [5,11]. Beyond serogroups W and Y, the only other risk factor associated with increased mortality was increasing age. This factor was also noted in a number of other studies [10,11,26]. The presence of meningitis vs meningococcaemia/septicaemia decreased the odds of death by 17 times. This finding was in agreement with similar findings in other population series and may be explained by an assumption that in the case of meningitis, the infection and resulting inflammation is restricted mainly to the nervous system and spares other tissues [11,26].

The main limitation of this study is its retrospective character. However, because of the questionnaire that was completed with clinical information and submitted to the USCCD when a new case was diagnosed, adequate information was available on all cases of IMD. Another limitation is that there were gaps in clinical data regarding mortality as clinical outcome in 11% of cases. A third limitation is the small number of IMD cases overall, which limited statistical power when risk factors for mortality were analysed and prohibited multi-variable regression analysis. These could explain why clinical parameters on admission such as the presence of confusion, the low or normal number of white blood cells or the short duration between onset of symptoms and admission to hospital could not be shown to be statistically significant risk factors for the outcome death, although they were shown to be associated with increased risk of death in other studies [11]. Another limitation which prevents us from fully assessing the impact of immunisation programmes is the dual scheme of immunisation providers which operated until early 2019, i.e. the public and private sector and the resulting differences in the availability of vaccines. It is, however, important to say that since 2019, both sectors of healthcare providers have been integrated under the National Health Service Organization. Vaccines are now determined by the National Immunisation committee and are given free of charge to all population.

## Conclusions

Although rare, IMD remains an important cause of morbidity and mortality among the Cypriot population. Introduction of vaccines, as well as appropriate preventive measures taken when a new case is diagnosed, have resulted in a decreased incidence of disease. It appears that serogroup B accounts for an important proportion of new cases especially in the high incidence age groups infants and adolescents. This occurred despite the fact that meningococcal B vaccine had already been offered by the private sector to a substantial yet unknown percentage of children. Serogroups Y and W have also become important as causes of IMD in the population and were the the main serogroups causing mortality. The introduction of MenACWY at the age of 12 months and also before joining the army is expected to decrease IMD caused by these serogroups.

Continuous monitoring of IMD in Cyprus remains an important tool in the prevention and control of this disease and will help in the evaluation of the immunisation programmes. National guidelines for the prevention, early recognition and management of the disease have to be prepared and disseminated to the healthcare providers as well as to the general public.

## References

[1] Cartwright KA Stuart JM Jones DM Noah ND The Stonehouse survey: nasopharyngeal carriage of meningococci and Neisseria lactamica. Epidemiol Infect. 1987;99(3):591-601. 10.1017/S0950268800066449 3123263 PMC2249239

[2] Public Health England (PHE). Meningococcal: the green book, chapter 22. London: PHE; 2016. Available from: https://www.gov.uk/government/publications/meningococcal-the-green-book-chapter-22

[3] Pace D Pollard AJ Meningococcal disease: clinical presentation and sequelae. Vaccine. 2012;30(Suppl 2):B3-9. 10.1016/j.vaccine.2011.12.062 22607896

[4] Harrison LH Trotter CL Ramsay ME Global epidemiology of meningococcal disease. Vaccine. 2009;27(Suppl 2):B51-63. 10.1016/j.vaccine.2009.04.063 19477562

[5] Whittaker R Dias JG Ramliden M Ködmön C Economopoulou A Beer N The epidemiology of invasive meningococcal disease in EU/EEA countries, 2004-2014. Vaccine. 2017;35(16):2034-41. 10.1016/j.vaccine.2017.03.007 28314560

[6] European Centre for Disease Prevention and Control (ECDC). Invasive meningococcal disease. In: ECDC. Annual epidemiological report for 2017. Stockholm: ECDC; 2019. Available from: https://www.ecdc.europa.eu/sites/default/files/documents/AER_for_2017-invasive-meningococcal-disease.pdf

[7] Kirsch EA Barton RP Kitchen L Giroir BP Pathophysiology, treatment and outcome of meningococcemia: a review and recent experience. Pediatr Infect Dis J. 1996;15(11):967-78, quiz 979. 10.1097/00006454-199611000-00009 8933544

[8] Trotter CL Chandra M Cano R Larrauri A Ramsay ME Brehony C A surveillance network for meningococcal disease in Europe. FEMS Microbiol Rev. 2007;31(1):27-36. 10.1111/j.1574-6976.2006.00060.x 17168995

[9] Darton T Guiver M Naylor S Jack DL Kaczmarski EB Borrow R Severity of meningococcal disease associated with genomic bacterial load. Clin Infect Dis. 2009;48(5):587-94. 10.1086/596707 19191644

[10] Cohn AC MacNeil JR Harrison LH Hatcher C Theodore J Schmidt M Changes in Neisseria meningitidis disease epidemiology in the United States, 1998-2007: implications for prevention of meningococcal disease. Clin Infect Dis. 2010;50(2):184-91. 10.1086/649209 20001736

[11] Sadarangani M Scheifele DW Halperin SA Vaudry W Le Saux N Tsang R Outcomes of invasive meningococcal disease in adults and children in Canada between 2002 and 2011: a prospective cohort study. Clin Infect Dis. 2015;60(8):e27-35. 10.1093/cid/civ028 25605282

[12] Campbell H Andrews N Borrow R Trotter C Miller E Updated postlicensure surveillance of the meningococcal C conjugate vaccine in England and Wales: effectiveness, validation of serological correlates of protection, and modeling predictions of the duration of herd immunity. Clin Vaccine Immunol. 2010;17(5):840-7. 10.1128/CVI.00529-09 20219881 PMC2863391

[13] Trotter CL Andrews NJ Kaczmarski EB Miller E Ramsay ME Effectiveness of meningococcal serogroup C conjugate vaccine 4 years after introduction. Lancet. 2004;364(9431):365-7. 10.1016/S0140-6736(04)16725-1 15276396

[14] Maiden MC Stuart JM UK Meningococcal Carraige Group Carriage of serogroup C meningococci 1 year after meningococcal C conjugate polysaccharide vaccination. Lancet. 2002;359(9320):1829-31. 10.1016/S0140-6736(02)08679-8 12044380

[15] Bröker M Bukovski S Culic D Jacobsson S Koliou M Kuusi M Meningococcal serogroup Y emergence in Europe: high importance in some European regions in 2012. Hum Vaccin Immunother. 2014;10(6):1725-8. 10.4161/hv.28206 24608912 PMC5396223

[16] Krone M Gray S Abad R Skoczyńska A Stefanelli P van der Ende A Increase of invasive meningococcal serogroup W disease in Europe, 2013 to 2017. Euro Surveill. 2019;24(14). 10.2807/1560-7917.ES.2019.24.14.1800245 30968827 PMC6462787

[17] Cohn AC MacNeil JR Clark TA Ortega-Sanchez IR Briere EZ Meissner HC Prevention and control of meningococcal disease: recommendations of the Advisory Committee on Immunization Practices (ACIP). MMWR Recomm Rep. 2013;62(RR-2):1-28. 23515099

[18] European Centre for Disease Prevention and Control (ECDC). Surveillance of invasive bacterial diseases in Europe, 2012. Stockholm: ECDC; 2015. Available from: https://ecdc.europa.eu/sites/portal/files/media/en/publications/Publications/Surveillance%20of%20IBD%20in%20Europe%202012.pdf

[19] Ladhani SN Flood JS Ramsay ME Campbell H Gray SJ Kaczmarski EB Invasive meningococcal disease in England and Wales: implications for the introduction of new vaccines. Vaccine. 2012;30(24):3710-6. 10.1016/j.vaccine.2012.03.011 22429756

[20] Parikh SR Andrews NJ Beebeejaun K Campbell H Ribeiro S Ward C Effectiveness and impact of a reduced infant schedule of 4CMenB vaccine against group B meningococcal disease in England: a national observational cohort study. Lancet. 2016;388(10061):2775-82. 10.1016/S0140-6736(16)31921-3 28100432

[21] Unit for surveillance and control of communicable diseases, Medical and public health services. National Vaccination Coverage survey in toddlers 17-24 months old, 2015. Nicosia: Cyprus Ministry of Health; 2015.

[22] Unit for Surveillance and Control of Communicable Diseases, Medical and Public Health Services. National Vaccination Coverage survey in toddlers 17-24 months old, 2009. Nicosia: Cyprus Ministry of Health; 2009.

[23] European Centre for Disease Prevention and Control (ECDC). Invasive meningococcal disease, In: ECDC Annual epidemiological report for 2016, Stockholm: ECDC; 2018. Available from: https://ecdc.europa.eu/en/publications-data/invasive-meningococcal-disease-annual-epidemiological-report-2016

[24] Statistical service of Cyprus (CYSTAT) – Ministry of Finance. Demographic Report 2017. Nicosia: CYSTAT; 2018 Available from: https://www.mof.gov.cy/mof/cystat/statistics.nsf/All/77A0741D88AEB4B7C225834E003F69F2/$file/Demographic_Report-2017-EN-301118.pdf?OpenElement

[25] European Commission. Commission Implementing Decision of 8 August 2012 amending Decision 2002/253/EC laying down case definitions for reporting communicable diseases to the Community network under Decision No 2119/98/EC of the European Parliament and of the Council. 2012. Available from: https://publications.europa.eu/en/publication-detail/-/publication/10ed460f-0711-11e2-8e28-01aa75ed71a1/language-en

[26] Piscopo T Mallia-Azzopordi C Grech V Muscat M Attard-Montalto S Mallia C Epidemiology and prognostic factors in meningococcal disease in a small island population: Malta 1994-1998. Eur J Epidemiol. 2000;16(11):1051-6. 10.1023/A:1010865315425 11421475

[27] Ó Maoldomhnaigh C Drew RJ Gavin P Cafferkey M Butler KM Invasive meningococcal disease in children in Ireland, 2001-2011. Arch Dis Child. 2016;101(12):1125-9. 10.1136/archdischild-2015-310215 27566800

[28] World Health Organization (WHO). Laboratory methods for the diagnosis of meningitis caused by Neisseria meningitidis, Streptococcus pneumoniae, and Haemophilus influenza. In: WHO Manual. 2nd ed. Chapter 7. WHO/ CDS/CSR/EDC/99.7. Geneva: WHO; 2011. Available from: https://www.cdc.gov/meningitis/lab-manual/full-manual.pdf

[29] Tzanakaki G Tsopanomichalou M Kesanopoulos K Matzourani R Sioumala M Tabaki A Simultaneous single-tube PCR assay for the detection of Neisseria meningitidis, Haemophilus influenzae type b and Streptococcus pneumoniae. Clin Microbiol Infect. 2005;11(5):386-90. 10.1111/j.1469-0691.2005.01109.x 15819865

[30] Drakopoulou Z Kesanopoulos K Sioumala M Tambaki A Kremastinou J Tzanakaki G Simultaneous single-tube PCR-based assay for the direct identification of the five most common meningococcal serogroups from clinical samples. FEMS Immunol Med Microbiol. 2008;53(2):178-82. 10.1111/j.1574-695X.2008.00406.x 18623625

[31] Maiden MC Bygraves JA Feil E Morelli G Russell JE Urwin R Multilocus sequence typing: a portable approach to the identification of clones within populations of pathogenic microorganisms. Proc Natl Acad Sci USA. 1998;95(6):3140-5. 10.1073/pnas.95.6.3140 9501229 PMC19708

[32] Russell JE Jolley KA Feavers IM Maiden MC Suker J PorA variable regions of Neisseria meningitidis. Emerg Infect Dis. 2004;10(4):674-8. 10.3201/eid1004.030247 15200858 PMC3323080

[33] Thompson EAL Feavers IM Maiden MCJ Antigenic diversity of meningococcal enterobactin receptor FetA, a vaccine component. Microbiology. 2003;149(Pt 7):1849-58. 10.1099/mic.0.26131-0 12855736

[34] Leber AL Everhart K Balada-Llasat JM Cullison J Daly J Holt S Multicenter evaluation of biofire filmarray meningitis/encephalitis panel for detection of bacteria, viruses, and yeast in cerebrospinal fluid specimens. J Clin Microbiol. 2016;54(9):2251-61. 10.1128/JCM.00730-16 27335149 PMC5005480

[35] R Core Team. R: A language and environment for statistical computing. Vienna: R Foundation for Statistical Computing; 2018. Available from: https://www.R-project.org/

[36] Wickham H. ggplot2: Elegant graphics for data analysis. New York: Springer; 2016.

[37] Heinze G, Ploner M, Dunkler D, Southworth H. logistf: Firth's bias-reduced logistic regression. R package version 1.23. 2018. Available from: https://CRAN.R-project.org/package=logistf

[38] Heinze G Schemper M A solution to the problem of separation in logistic regression. Stat Med. 2002;21(16):2409-19. 10.1002/sim.1047 12210625

[39] National Immunisation Advisory Committee . Immunisation guidelines. Chapter 13 – Meningococcal Infection. Dublin: Health Service Executive (HSE); 2019. Available from: https://www.hse.ie/eng/health/immunisation/hcpinfo/guidelines/chapter13.pdf

[40] MacNeil JR Bennett N Farley MM Harrison LH Lynfield R Nichols M Epidemiology of infant meningococcal disease in the United States, 2006-2012. Pediatrics. 2015;135(2):e305-11. 10.1542/peds.2014-2035 25583921 PMC4803024

[41] Dunlop KA Coyle P Mitchell S Fairley D O’Neill H Jackson P Molecular testing of respiratory swabs aids early recognition of meningococcal disease in children. Diagn Microbiol Infect Dis. 2011;70(4):427-34. 10.1016/j.diagmicrobio.2011.03.018 21658876

[42] Judelsohn R Marshall GS The burden of infant meningococcal disease in the United States. J Pediatric Infect Dis Soc. 2012;1(1):64-73. 10.1093/jpids/pir003 23687573 PMC3656548

[43] European Centre for Disease Prevention and Control (ECDC). Expert opinion on the introduction of the meningococcal B (4CMenB) vaccine in the EU/EEA. Stockholm: ECDC; 2017. Available from: https://ecdc.europa.eu/en/publications-data/expert-opinion-introduction-meningococcal-b-4cmenb-vaccine-eueea

[44] American College Health Association (ACHA). Immunization recommendations for college students. Silver Spring: ACHA; 2018. Available from: https://www.acha.org/documents/resources/guidelines/ACHA_Immunization_Recommendations_Oct2018.pdf

[45] Public Health England (PHE). MenACWY: Information on vaccination for first time university entrants. London: PHE; 2015. Available from: https://assets.publishing.service.gov.uk/government/uploads/system/uploads/attachment_data/file/443670/MenACWY_vaccination_university_letter_FINAL08_07_15.pdf

[46] Maclean IH Bevan CE Observations on an epidemic of cerebrospinal meningitis in Cyprus and the record of a prophylactic experiment: (Section of Epidemiology and State Medicine). Proc R Soc Med. 1939;32(12):1551-72. 10.1177/003591573903201201 19992142 PMC1998102

[47] Jacobs JH Viboud C Tchetgen ET Schwartz J Steiner C Simonsen L The association of meningococcal disease with influenza in the United States, 1989-2009. PLoS One. 2014;9(9):e107486. 10.1371/journal.pone.0107486 25265409 PMC4180274

[48] Rameix-Welti MA Zarantonelli ML Giorgini D Ruckly C Marasescu M van der Werf S Influenza A virus neuraminidase enhances meningococcal adhesion to epithelial cells through interaction with sialic acid-containing meningococcal capsules. Infect Immun. 2009;77(9):3588-95. 10.1128/IAI.00155-09 19528219 PMC2738041

